# Torque Teno Sus Virus (TTSuV) in Cell Cultures and Trypsin

**DOI:** 10.1371/journal.pone.0017501

**Published:** 2011-03-02

**Authors:** Thais Fumaco Teixeira, Diogenes Dezen, Samuel Paulo Cibulski, Ana Paula Muterle Varela, Carine Lidiane Holz, Ana Cláudia Franco, Paulo Michel Roehe

**Affiliations:** 1 Equipe de Virology, FEPAGRO Animal Health - Instituto de Pesquisas Veterinárias Desidério Finamor (IPVDF), Eldorado do Sul, Rio Grande do Sul, Brazil; 2 Virology Laboratory, Department of Microbiology, Immunology and Parasitology, Instituto de Ciências Básicas da Saúde, Universidade Federal do Rio Grande do Sul, Porto Alegre, Rio Grande do Sul, Brazil; 3 CIRAD, Départament Systèmes Biologiques, UR-15, Campus International de Baillarguet, Montpellier, France; University of Minnesota, United States of America

## Abstract

Torque teno sus virus (TTSuV), a member of the family *Anelloviridae*, is a single-stranded, circular DNA virus, widely distributed in swine populations. Presently, two TTSuV genogroups are recognized: *Torque teno sus virus 1* (TTSuV1) and *Torque teno sus virus 2* (TTSuV2). TTSuV genomes have been found in commercial vaccines for swine, enzyme preparations and other drugs containing components of porcine origin. However, no studies have been made looking for TTSuV in cell cultures. In the present study, a search for TTSuV genomes was carried out in cell culture lineages, in sera used as supplement for cell culture media as well as in trypsin used for cell disaggregation. DNA obtained from twenty-five cell lineages (ten from cultures in routine multiplication and fifteen from frozen ampoules), nine samples of sera used in cell culture media and five batches of trypsin were examined for the presence of TTSuV DNA. Fifteen cell lineages, originated from thirteen different species contained amplifiable TTSuV genomes, including an ampoule with a cell lineage frozen in 1985. Three cell lineages of swine origin were co-infected with both TTSuV1 and TTSuV2. One batch of trypsin contained two distinct TTSuV1 plus one TTSuV2 genome, suggesting that this might have been the source of contamination, as supported by phylogenetic analyses of sequenced amplicons. Samples of fetal bovine and calf sera used in cell culture media did not contain amplifiable TTSuV DNA. This is the first report on the presence of TTSuV as contaminants in cell lineages. In addition, detection of the viral genome in an ampoule frozen in 1985 provides evidence that TTSuV contamination is not a recent event. These findings highlight the risks of TTSuV contamination in cell cultures, what may be source for contamination of biological products or compromise results of studies involving *in vitro* multiplied cells.

## Introduction

Torque teno viruses (TTVs) are small, non-enveloped viruses that contain a circular single-stranded DNA genome of negative polarity [1], presently classified in the family *Anelloviridae* [2]. TTVs were first detected in 1997 in a Japanese patient with post-transfusion hepatitis of unknown etiology [3]. Since then, other human TTVs have been described with distinct genome sizes; Torque teno “midi viruses” (TTMDV) comprises viruses with genomes sizes with about 3.2 kb [4], whereas Torque teno “mini viruses” (TTMV) have genome sizes between 2.8 kb and 2.9 kb [5]. TTVs are not restricted to human hosts and have also been identified in a number of other species, including non-human primates, tupayas, cats, dogs, pigs, chickens, cows and sheep [1], [6]–[11].

In swine, two distinct genogroups, *Torque teno sus virus 1* (TTSuV1) and *Torque teno sus virus 2* (TTSuV2), have been identified [1], [7], [12]. Torque teno sus viruses (TTSuVs) are widely distributed in swine populations, though reported prevalences are quite variable [13]–[16]. The association of TTSuVs with disease is currently subject of studies; data suggest that TTSuVs may participate as coadjuvants in other pathological conditions of swine, such as post-weaning multisystemic syndrome (PMWS) and porcine dermatitis and nefropathy syndrome (PDNS), diseases primarily associated to porcine circovirus type 2 infections [13], [17]–[18].

TTSuVs have also been detected in colostrum and in stillborns, suggesting vertical transmission of the virus [19]. The finding of TTSuV genomes in semen of boars indicates that the virus may possibly be transmitted by natural or artificial reproduction [20]. Others have raised the possibility of TTSuV transmission by contaminated biological products, since TTSuVs genomes have been identified in commercial vaccines for swine and in enzyme preparations and other drugs formulated with components of porcine origin [21]. This possibility, however, awaits further investigation.

We have been attempting to propagate TTSuV *in vitro* cultured cells. However, this would require previous testing of cells and media to ensure that no preexisting contamination would undermine virus isolation. To date, no previous data on the presence of TTSuV in cell cultures is available. In view of that, a search was made for TTSuV genomes in a number of available established cell lineages. In addition, other frequent sources of cell culture contaminants, such as fetal calf sera and trypsin used routinely in cell culture manipulation were also tested for the presence of TTSuV.

## Results

A duplex PCR was designed to amplify genome fragments of both TTSuV1 and TTSuV2 in a same reaction. The sensitivity of the duplex PCR was determined by performing the reaction with different concentrations of DNA extracted from pCR2.1 plasmid containing TTSuV1 or TTSuV2 PCR products. The minimum number of TTSuV copies that could be identified with this method was determined by testing tenfold dilutions of plasmid DNA in the duplex PCR. With this approach, it was determined that the lowest number of genome molecules detectable by the assay was 100 molecules of TTSuV1 and 1000 molecules of TTSuV2 per reaction.

Once the sensitivity of the tests was determined, the search for the presence of TTSuV contamination in cell cultures and related products was carried out. The results of these findings are summarized on Table 1. Fifteen cell culture lineages tested contained amplifiable TTSuV1 and/or TTSuV2 genomes, including cells that were tested as soon as thawed out of the liquid nitrogen. Some cell culture lineages of swine origin (PK15 PCV1 free, ST and PK15) were co-infected with both TTSuV1 and TTSuV2. All samples from sera that had been used as cell culture media supplement resulted negative for the presence of amplifiable TTSuV DNA. One batch of trypsin contained genomes of two distinct TTSuV1 as well as TTSuV2. This batch of trypsin was in use on the ten cell lineages that were currently being multiplied in the laboratory. These were found to be contaminated with either TTSuV1 or TTSuV2. The three cell lineages of porcine origin, on which the same trypsin batch was also been used, was found contaminated with both types of TTSuV. The other four batches of trypsin tested did not contain amplifiable TTSuV DNA (Table 1).

**Table 1:** Detection of genomes of *Torque teno sus virus 1* (TTSuV1) and *2* (TTSuV2) in cell lineages, sera and trypsin batches.

Cell lineages, serum and trypsin	Origin	N° of Passages#	Presence of viral DNA of TTSuV1	Presence of viral DNA of TTSuV2
Baby hamster kidney (BHK-21)	A. Lutz^a^	75	−	+
Chicken embryo related (CER)	VLA^b^	130	−	+
Crandell feline kidney (CrFK)	Unk^c^	241	−	+
Human embryonic intestine (H407)	UFRGS^d^	19	+	−
Human leukemic cell (K562) (28/05/04)*	UFRGS	7	+	−
African green monkey kidney embryonic (MA-104) (28/04/93)*	UFSM^e^	34	+	−
Madin-Darby bovine kidney (MDBK) (17/08/01)*	Panaftosa^f^	129	+	−
Madin-Darby canine kidney (MDCK) (25/10/05)*	Unicamp^g^	60	+	−
Porcine kidney PK15	UFSM	26	+	+
Porcine kidney (PK-2a)	VLA	38	−	+
Porcine kidney PK15 PCV1 free (PKsC3)	Cloned from PK15 at IPVDF	35	+	+
Swine kidney (SK6)	VLA	120	−	+
Swine testicle (ST)	Embrapa^h^	56	+	+
Bovine thyroid cell (TB) (27/02/85)*	Flow^i^	13	+	−
African green monkey kidney (Vero)	Fiocruz^j^	118	−	+
Canine Carcinoma (A-72) (2/07/08)*	VLA	45	−	−
Mutant MDBK Resistant to BVDV Infection (CRIB) (16/01/06)*	UFSM	120	−	−
Embryonic Bovine Trachea (EBTr) (29/12/04)*	IPVDF^k^	30	−	−
Equine Dermis (ED) (9/07/08)*	UFPEL^l^	17	−	−
Foetal Lamb Kidney (FLK) (6/12/90)*	UFPEL	137	−	−
Murine Fibrosarcoma (L929) (3/06/08)*	UFRJ^m^	10	−	−
Monkey Kidney (LLC-MK_2_) (15/07/86)*	UFRJ	61	−	−
Murine Neuroblastoma (N2A) (18/10/04)*	VLA	202	−	−
Rabbit Kidney (RK13) (13/01/93)*	UFPEL	54	−	−
Murine myeloma (SP2/O-Ag14)	VLA	34	−	−
Fetal Bovine Serum	Manufacturer A	na§	−	−
Fetal Bovine Serum	Manufacturer B	na	−	−
Fetal Bovine Serum	Manufacturer C	na	−	−
Calf Serum (treated in house with polyethylene glycol)	IPVDF	na	−	−
Calf serum	IPVDF	na	−	−
Horse serum (inactivated)	IPVDF	na	−	−
Horse serum (1 donor)	IPVDF	na	−	−
Horse serum (pool)	IPVDF	na	−	−
Ovine serum	IPVDF	na	−	−
Trypsin	Manufacturer A	na	+	+
Trypsin	Manufacturer B	na	−	−
Trypsin	Manufacturer C	na	−	−
Trypsin	Manufacturer D	na	−	−
Trypsin	Manufacturer E	na	−	−

*Date of ampouling/freezing in liquid nitrogen;

a Adolfo Lutz Institute, Brazil;

b Veterinary Laboratories Agency, Weybridge, UK;

c Unknown origin;

d Federal University of Rio Grande do Sul, Brazil;

e Federal University of Santa Maria, Brazil;

f Panaftosa;

g Campinas University, Brazil;

h Empresa Brasileira de Pesquisa Agropecuária, Brazil;

i Flow Laboratories, USA;

j Fundação Oswaldo Cruz, Brazil.

k Prepared at Fepagro Saúde Animal – IPVDF;

l Federal University of Pelotas, RS, Brazil;

m Federal University of Rio de Janeiro, RJ, Brazil;

# refers to the number of passages continuing the sequencial passage number as received from the source;

§ not applicable.

Amplicons with the expected size (107 bp for TTSuV1 and 103 bp for TTSuV2) were excised from 1% agarose gels, cloned and sequenced. Twenty one nucleotide sequences corresponding to such amplicons were submitted to GenBank (accession numbers GU574709 to GU574729).

**Figure 1:**
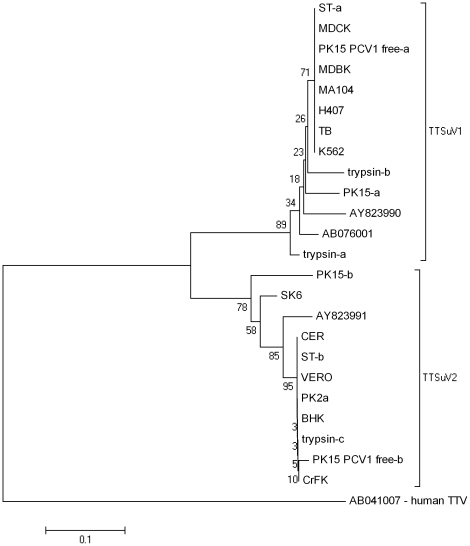
Neighbour-joining phylogenetic tree constructed based on the nucleotide sequences of the noncoding region of TTSuV genomes. Bootstrap values are indicated above major branches. AB076001 and AY823990 are reference sequences for TTSuV1 and AY823991 is the reference strains for TTSuV2. Small letters (−a,−b,−c) after names refer to different sequences identified in a particular cell lineage (or trypsin batch).

The phylogenetic tree (Figure 1) inferred by the neighbor-joining method allowed the grouping of virus genomes in TTSuV1 and TTSuV2 genogroups. Eleven sequences were clustered within the TTSuV1 genogroup, displaying between 88.71% to 100% sequence similarity to the reference strains in genogroup 1 (AB076001, AY823990). Ten other sequences clustered within the TTSuV2 genogroup, with 83.79% to 100% sequence similarity to the reference strain (AY823991). TTSuV1 genomes identified in trypsin-b were nearly identical to those found in eight of the contaminated cells and TTSuV2 genome detected in trypsin-c was nearly identical to those found in seven cells showing that maybe these cells can be contaminated by residual trypsin. It can also be seen that sequences from PK15-b and SK6 lineages were the most filogenetically distant sequences within the TTSuV2 genogroup, suggesting either a different source of contamination, or that the original virus sequence had been mutated during replication.

## Discussion

Koch's postulates are being once more challenged by molecular methods of genome detection. Diagnostic methods have evolved in such a way that in many instances the genome of an agent can be identified without the need for its previous isolation. While searching for DNA- containing agents that may be infecting swine - regardless of any association with disease - using methods that allow genome amplification without previous knowledge of nucleotide sequences [6], [7] our group identified TTSuV contamination in farming pigs [22]. In order to proceed on the study of such agents, a natural development was to try to multiply such viruses in cell cultures. However, this would require cultured cells free of TTSuV contamination. Thus, the present study was set up to examine whether the cells available in our laboratory would be contaminated. As result of this search, TTSuV genomes were detected in cell lineages of porcine and non-porcine origin. Indeed, fifteen out of the 25 cells tested revealed TTSuV contamination. Three of the cell lineages of porcine origin were infected with both TTSuV1 and TTSuV2.

Once contamination was detected in cultured cells, the identification of the source of contamination was imperative. One batch of trypsin was contaminated with two distinct variants of TTSuV1 as well as with TTSuV2. The other trypsin batches tested were negative for the presence of TTSuV. The sera used as media supplement was not found to contain TTSuV, a result which might be expected, since all sera were of non-porcine origin. Therefore, these findings were pointing towards the contaminated batch of trypsin as source of TTSuV contamination. Phylogenetic analyses suggest that the TTSuV genomes detected in most cell lineages were closely related - but not identical - to those detected in the contaminated batch of trypsin. Therefore, this seems in fact the most likely source for contamination of cultures. This batch of trypsin was being used on all cells (BHK-21, CER, CrFK, H407, PK15, PK-2 nd, PK15 PCV1 free, SK6, ST, and Vero) being multiplied in the laboratory at the time this study was being carried out. All these cells were found to contain at least one TTSuV variant. Clearly, the finding of TTSuV genomes in cells treated with contaminated trypsin does not ensure that virus multiplication took place in such cells. Indeed, it may be argued that virus carried over by residual trypsin might have been the source of TTSuV contamination for at least some of the cells. However, if this was the case, all infected cells should reveal contamination with both types of TTSuV detected in the contaminated trypsin. Moreover, the nucleotide sequences of the recovered fragments should be very similar, which was not the case. In fact, phylogenetic analysis shows that, although some sequences are indeed very similar, others are quite phylogenetically apart, indicating that either contamination originated from distinct sources, or the original virus had undergone distinct evolutionary pathways during replication. As an example, the phylogenetic distance between the TTSuV2 fragments from SK6 and PK15-b cells (Figure 1) suggests these viruses probably originated from distinct sources - perhaps another batch of contaminated trypsin used in the past, or yet the tissues from which cells were originally prepared. In such cases, however, the precise source of contamination can only be guessed with the data here available.

Despite that, there still remains the possibility that some of the cells were in fact carrying virus from residual trypsin. The sensitivity threshold of the PCR employed in this study was 100–1000 molecules of viral DNA, indicating that relatively high viral loads were needed to be detected in the cell lineages. In addition, in attempting to minimize chances of amplifying viral genomes that could be present in residual trypsin, the supernatant medium was carefully washed out with PBS three times before DNA extractions. However, the possibility of residual trypsin contamination carry over cannot be fully discarded and must remain as an additional risk to be considered when searching for anelloviruses in cell cultures.

It must also be reminded that other TTSuV variants could be present in cells and trypsin and might have remained undetected by the method employed here; this possibility also cannot be completely ruled out. In view of the specificity of the primers designed for this study, these would not be detected. Likewise, it is also possible - and quite probable, in our belief, based on the apparently wide dispersion of anelloviruses in nature - that sera may act as a potential source for anelloviruses derived from other animal species. This might eventually lead to infection of other cultured cell lineages. However, this must also be taken into account when dealing with cultured cells.

Interestingly, from the results obtained here, it became apparent that TTSuV contamination of cultured cells is not a recent event. A cell lineage that had been ampouled in 1985 and was tested as soon as thawed out of the liquid nitrogen was also found to contain TTSuV1. Therefore, such viruses have been circulating for at least more than 25 years. This adds to the evidence for the circulation of such viruses, as indeed detected in a retrospective study on swine sera collected in the same year, revealing that TTSuV1 and TTSuV2 were already detected in the original source species [23].

Knowledge on TTSuV- as well as on anelloviruses in general - is still in its early days; clear association between these viruses and disease has not yet been fully established. It is possible that TTSuV might act as incidental pathogens, where disease would become evident only under exceptional circumstances. In some infections, the viral load is a critical for the development of disease. It has been suggested that anelloviruses might be comensal agents under normal circumstances, incapable of exceeding the threshold of a disease-causing load [24]. Also interesting is the observation that anelloviruses may be able to impair replication of other viruses. An association was detected between a higher prevalence of TTSuV1 in healthy, non-PMWS-affected pigs, than in PMWS-affected animals [22]. In this sense, anelloviruses might somehow bring some benefit the host, an aspect hitherto unexplored [25].

In addition, the possibility of xenotransplantation of swine tissues to humans would require that no adventitious agents are present in tissues of potential donors to ensure no contamination of transplant recipients [26]. Therefore, TTSuV contamination must be examined in light of such possibility.

Whichever is the case, appropriate measures should be taken to ensure that no TTSuV contamination occurs through the usage of contaminated cell culture or the reagents used for *in vitro* cell multiplication and maintenance. Further studies should be conducted to confirm whether TTSuV might give rise to productive infections in non-porcine cell lineages.

## Materials and Methods

### Cells, sera and trypsin

Twenty-five cell lines (ten from cultured cells and fifteen from ampoules stocked in liquid nitrogen) obtained from the laboratory cell bank were used in the experiments (Table 1). Cell culture multiplication was performed following standard methods [27]. Cell lineages were multiplied in Eagle's minimal essential medium (MEM) supplemented with 10% fetal bovine serum and antibiotics (penicillin 100 IU/mL; streptomycin 100 µg/mL). In addition, nine different batches of sera from different species [bovine (05), equine(3), ovine(1)] used as supplements to cell culture media in different moments in the cell culture laboratory, as well as and five batches of trypsin from different manufacturers were included in this study (Table 1).

### DNA extraction

DNA extraction from cultured cells was performed as follows: the culture medium was removed and the confluent monolayer washed with PBS (0.15 M NaCl, 0.07 M Na_2_HPO_4_, 8 mM NaH_2_PO_4_, pH 7.4). The PBS was discarded and 4 ml of lysis buffer [20 mM Tris–HCl (pH 8.0); 2 mM EDTA (pH 8.0), 300 mM NaCl; 100 µg proteinase K, 1% sodium duodecyl sulphate (SDS)] were added to flasks and incubated for 90 minutes at 37°C. Subsequently, 500 µl of the digested material were transferred to new tubes. The DNA was extracted with phenol and after with chloroform/isoamyl alcohol (24∶1) [28]. The DNA was precipitated with ethanol, the pellet dried and resuspended in 50 µL TE (10 mM Tris, 1 mM EDTA, pH 7.4) containing 20 µg/mL RNase A. DNA extraction from cells thawed from liquid nitrogen was carried out as follows: ampoules were thawed and centrifuged for 1 min at 9,000×*g*. The supernatant was removed and the cell pellet resuspended in 500 µL of PBS, 3% SDS, 200 µg/ml proteinase K and incubated for 90 minutes at 37°C. The DNA was extracted as mentioned above. The sera DNA extraction was performed with 500 µL of serum and the trypsin DNA extraction was performed with 50 mg of trypsin diluted in 500 µL Milli-Q water. The DNA was extracted with phenol/chloroform/isoamyl alcohol as mentioned above. DNA from samples was quantified with known amounts of lambda/*Hin*d III DNA as standard in 1% agarose gels, stained with ethidium bromide and visualized on a UV source. To avoid cross-contamination, DNA extraction was performed in different days, with each cell line being processed separately and with filter tips; after each extraction, laminar flow cabinets were cleaned with ethanol and UV-sterilized for at least 30 minutes before working with another cell lineage. No more than three cell lineages were processed on a same working day.

### Detection of TTSuV

To detect simultaneously TTSuV1 and TTSuV2, a duplex PCR was designed. PCR primers were based on sequences available at GenBank (AB076001– AY823991) and were designed to amplify the non-coding regions of TTSuV1 and TTSuV2. Two forward primers and one common reverse primer were designed: primer “forward-1” (5′ GGG AGC TCA AGT CCT CAT TTG 3′) and a common reverse primer (5′ GCG GCA TAA ACT CAG CCA TTC 3′) targeted a 107 bp DNA fragment (nucleotide positions 221–328 on TTSuV1 genome), whereas primer “forward-2” (5′ GGG CCW GAA GTC CTC ATT AG 3′) plus the common reverse primer were expected to amplify a 103 bp fragment (nucleotide positions 170–273 on TTSuV2 genome). The PCR was carried out in 25 µL volumes with contained 2 µL of DNA (100 ng), 5 pmol primer forward-1, 5 pmol primer forward-2, 5 pmol primer reverse, 0.8 mM dNTP, 1.5 mM MgCl_2_ and 1 U Taq DNA polymerase (Invitrogen). The PCR program consisted of an initial reaction at 94°C for 3 min, followed by 35 cycles at 94°C (30 s), 65°C (30 s) and 72°C (30 s), with a final extension period of 10 min at 72°C. Amplicons were electrophoresed in 1% agarose gel and purified using a commercial kit (GFX™ Purification Kit; Amersham Biosciences).

All PCR products were cloned into plasmids using a TA cloning strategy (pCR 2.1 TOPO Cloning, Invitrogen). At least three recombinant plasmids of each reaction were sequenced on both strands using M13-forward and M13-reverse oligonucleotides as primers in a MegaBACE 500 apparatus with the Dyenamic ET terminator cycle sequencing kit (Amersham Biosciences) following the manufacturer's protocol. Sequence identification was performed using NCBI nucleotide BLAST searches (http://blast.ncbi.nlm.nih.gov/Blast.cgi).

To avoid contamination, filter tips were used to prepare the PCR reactions and separate rooms were used to prepare reaction buffers, to extract DNA, and to examine PCR products. A negative control (with ultra pure water instead of sample DNA) was included in every ten PCR tubes as additional contamination controls. Positive controls consisted of reactions with cloned TTSuV1 and TTSuV2 DNA (see below).

### Sensitivity assay

In order to determine the PCR sensitivity, amplicons from TTSuV1 (107 bp) and TTSuV2 (103 bp) were cloned into plasmids as described above. The sensitivity of the PCR was determined by amplification of tenfold dilutions of known amounts of each plasmid DNA in the duplex PCR. These experiments were repeated three times. The same plasmids were also used as positive controls in the duplex PCR assays.

### Phylogenetic analyses

The obtained sequences were aligned with two sequences proposed as TTSuV1 prototypes (accession no. AY823990 and AB076001) and one sequence proposed as TTSuV2 prototype (accession no. AY823991) available at GenBank [1], [7]. A human TTV sequence was included in the alignment as outgroup (accession no. AB041007). Sequences were aligned using the ClustalW program within the MEGA 4 package. The construction of phylogenetic tree was carried out using the neighbor-joining (NJ) method in the MEGA 4 software package, based on Kimura two-parameter distance estimation method. Bootstrap resampling was performed for each analysis (1000 replications).

## References

[1] Okamoto H, Takahashi M, Nishizawa T, Tawara A, Fukai K (2002). Genomic characterization of TT viruses (TTV) in pigs, cats and dogs and their relatedness with species-specific TTV in primates and tupaias.. J Gen Virol.

[2] Biagini P, Todd D, Bendinelli M, Hino S, Mankertz A, Fauquet CM, Mayo MA, Maniloff J, Desselberger U, Ball LA (2005). Anellovirus.. Virus Taxonomy. Eighth Report of the International Committee on Taxonomy of Viruses.

[3] Nishizawa T, Okamoto H, Konishi K, Yoshizawa H, Miyakawa Y (1997). A novel DNA virus (TTV) associated with elevated transaminase levels in posttransfusion hepatitis of unknown etiology.. Biochem Biophys Res Commun.

[4] Ninomiya M, Takahashi M, Shimosegawa T, Okamoto H (2007). Analysis of the entire genomes of fifteen torque teno midi virus variants classifiable into a third group of genus Anellovirus.. Arch Virol.

[5] Takahashi K, Iwasa Y, Hijikata M, Mishiro S (2000). Identification of a new human DNA virus (TTV-like mini virus, TLMV) intermediately related to TT virus and chicken anemia virus.. Arch Virol.

[6] Biagini P, Uch R, Belhouchet M, Attoui H, Cantaloube JF (2007). Circular genomes related to anelloviruses identified in human and animal samples by using a combined rolling-circle amplification/sequence independent single primer amplification approach.. J Gen Virol.

[7] Niel C, Diniz-Mendes L, Devalle S (2005). Rolling-circle amplification of Torque teno virus (TTV) complete genomes from human and swine sera and identification of a novel swine TTV genogroup.. J Gen Virol.

[8] Okamoto H, Nishizawa T, Tawara A, Peng Y, Takahashi M (2000). Species-specific TT viruses in humans and nonhuman primates and their phylogenetic relatedness.. Virology.

[9] Okamoto H, Nishizawa T, Takahashi M, Tawara A, Peng Y (2001). Genomic and evolutionary characterization of TT virus (TTV) in tupaias and comparison with species-specific TTVs in humans and nonhuman primates.. J Gen Virol.

[10] Brassard J, Gagne MJ, Lamoureux L, Inglis GD, Leblanc D (2008). Molecular detection of bovine and porcine Torque teno virus in plasma and feces.. Vet Microbiol.

[11] Leary TP, Erker JC, Chalmers ML, Desai SM, Mushahwar IK (1999). Improved detection systems for TT virus reveal high prevalence in humans, non-human primates and farm animals.. J Gen Virol.

[12] Martínez L, Kekarainen T, Sibila M, Ruiz-Fons F, Vidal D (2006). Torque teno virus (TTV) is highly prevalent in the European wild boar (Sus scrofa).. Vet Microbiol.

[13] Kekarainen T, Sibila M, Segalés J (2006). Prevalence of swine Torque teno virus in post-weaning multisystemic wasting syndrome (PMWS)-affected and non-PMWS-affected pigs in Spain.. J Gen Virol.

[14] McKeown NE, Fenaux M, Halbur PG, Meng XJ (2004). Molecular characterization of porcine TT virus, an orphan virus, in pigs from six different countries.. Vet Microbiol.

[15] Bigarré L, Beven V, de Boisséson C, Grasland B, Rose N (2005). Pig anelloviruses are highly prevalent in swine herds in France.. J Gen Virol.

[16] Martelli F, Caprioli A, Di Bartolo I, Cibin V, Pezzotti G, Ruggeri FM, Ostanello F (2006). Detection of swine torque teno virus in Italian pig herds.. J Vet Med.

[17] Ellis JA, Allan G, Krakowka S (2008). Effect of coinfection with genogroup 1 porcine torque teno virus on porcine circovirus type 2-associated postweaning multisystemic wasting syndrome in gnotobiotic pigs.. Am J Vet Res.

[18] Krakowka S, Hartunian C, Hamberg A, Shoup D, Rings M (2008). Evaluation of induction of porcine dermatitis and nephropathy syndrome in gnotobiotic pigs with negative results for porcine circovirus type 2.. Am J Vet Res.

[19] Martínez-Guinó L, Kekarainen T, Segalés J (2009). Evidence of Torque teno virus (TTV) vertical transmission in swine.. Theriogenolgy.

[20] Kekarainen T, Lopez-Soria S, Segalés J (2007). Detection of swine Torque teno virus genogroups 1 and 2 in boar sera and semen.. Theriogenology.

[21] Kekarainen T, Martínez L, Segalés J (2009). Swine torque teno virus detection in pig commercial vaccines, enzymes for laboratory use and human drugs containing components of porcine origen.. J Gen Virol.

[22] Teixeira T (2008). Detection of posible agents asociated to porcine circovirus infections..

[23] Segalés J, Martínez-Guinó L, Cortey M, Navarro N, Huerta E (2009). Retrospective study on swine Torque teno virus genogroups 1 and 2 infection from 1985 to 2005 in Spain.. Vet Microbiol.

[24] Griffiths P (1999). Time to consider the concept of a commensal virus?. Rev Med Virol.

[25] Okamoto H (2009). History of discoveries and pathogenicity of TT viruses.. Curr Top Microbiol Immunol.

[26] Scobie L, Takeuchi Y (2009). Porcine endogenous retrovirus and other viruses in xenotransplantation.. Curr Opin Organ Transplant.

[27] Freshney RI (1992). Animal Cell Culture: A practical approach..

[28] Sambrook J, Russel DW (2001). Molecular Cloning: A Laboratory Manual (3^rd^ ed.).

